# Digital Variance Angiography in Selective Lower Limb Interventions

**DOI:** 10.1016/j.jvir.2021.09.024

**Published:** 2022-02

**Authors:** Rohit P. Thomas, Moritz B. Bastian, Simon Viniol, Alexander M. König, Sandeep S. Amin, Osama Eldergash, Johannes Schnabel, Marcell Gyánó, Dávid Szöllősi, István Góg, János P. Kiss, Szabolcs Osváth, Krisztián P. Szigeti, Andreas H. Mahnken

**Affiliations:** a Department of Diagnostic and Interventional Radiology, University Hospital Marburg, Philipps University of Marburg, Marburg, Germany; b Department of Diagnostic and Interventional Radiology, Klinikum Oldenburg AöR, Oldenburg, Germany; c Department of Interventional Radiology, Heart and Vascular Center, Semmelweis University, Budapest, Hungary; d Kinepict Health Ltd, Budapest, Hungary; e Department of Biophysics and Radiation Biology, Heart and Vascular Center, Semmelweis University, Budapest, Hungary; f Department of Vascular and Endovascular Surgery, Heart and Vascular Center, Semmelweis University, Budapest, Hungary

**Keywords:** CO_2_, carbon dioxide, CNR, contrast-to-noise ratio, DSA, digital subtraction angiography, DVA, digital variance angiography, ICM, iodinated contrast media, ROI, region of interest

## Abstract

**Purpose:**

To evaluate the potential benefits of digital variance angiography (DVA) in selective lower limb angiography and to compare the performance of 2 DVA algorithms (conventional DVA1 and the recently developed DVA2) to that of digital subtraction angiography (DSA).

**Materials and Methods:**

From November 2019 to May 2020, 112 iodinated contrast media (ICM) and 40 carbon dioxide (CO_2_) angiograms were collected from 15 and 13 peripheral artery disease patients, respectively. The DVA files were retrospectively generated from the same unsubtracted source file as DSA. The objectively calculated contrast-to-noise ratio (CNR) and the subjective visual image quality of DSA, DVA1, and DVA2 images were statistically compared using the Wilcoxon signed-rank test. The images were evaluated by 6 radiologists (R.P.T., S.V., A.M.K., S.S.A., O.E., and J.S.) from 2 centers using a 5-grade Likert scale.

**Results:**

Both DVA algorithms produced similar increase (at least 2-fold) in CNR values (*P* < .001) and significantly higher image quality scores than DSA, independent of the contrast agent used. The overall scores with ICM were 3.61 ± 0.05 for DSA, 4.30 ± 0.04 for DVA1, and 4.33 ± 0.04 for DVA2 (each *P* < .001 vs DSA). The scores for CO_2_ were 3.10 ± 0.14 for DSA, 3.63 ± 0.13 for DVA1 (*P* < .001 vs DSA), and 3.38 ± 0.13 for DVA2 (*P* < .05 vs DSA).

**Conclusions:**

DVA provides higher CNR and significantly better image quality in selective lower limb interventions irrespective of the contrast agent used. Between DVA algorithms, DVA1 is preferred because of its identical or better image quality than DVA2. DVA can potentially help the interventional decision process and its quality reserve might allow dose management (radiation/ICM reduction) in the future.

**Study Details**

**Study type:** Retrospective, observational, descriptive study

**Study phase:** Pilot study

**Research Highlights**

• In contrast to digital subtraction angiography (DSA), digital variance angiography (DVA) does not use a mask but calculates the standard deviation, variance, and other time-derived parameters of the X-ray attenuation for each pixel in an unsubtracted image series, resulting in the suppression of noise and high image quality.
• This study evaluated 2 different DVA algorithms in selective lower limb angiography using iodinated and carbon dioxide contrast media, using unsubtracted source files from DSA.
• Compared with DSA, DVA provided higher contrast-to-noise ratio and better image quality irrespective of contrast agent used. The conventional DVA algorithm yielded better image quality than the new algorithm.

Minimally invasive endovascular interventions have evolved to be the treatment of choice in cardiovascular disorders over the last several decades. These interventions require adequate imaging of the blood vessels, and good quality angiography plays a vital role in the decision-making process. Conventionally, the blood vessels are made visible during angiography with the injection of contrast agents (iodinated contrast media [ICM] or carbon dioxide [CO_2_]) and applied X-ray radiation. The acquired image data are subsequently processed with the technique of digital subtraction angiography (DSA) (1, 2, 3). ICM and CO_2_ are the standard contrast agents used for angiographic guidance. CO_2_, being a negative contrast, serves as an alternative when contraindications for ICM, such as allergy, renal insufficiency, or thyrotoxicosis, exist (4, 5, 6). Ongoing research is exploring the possibility of reducing the radiation dose and the amount of contrast agent used; however, these manipulations drastically impair the image quality (7, 8, 9, 10). Noise reduction algorithms provide a possible solution for this problem; however, their effectiveness is limited (11,12).

Digital variance angiography (DVA), a new technology based on kinetic imaging, is described to perform information extraction from images generated by penetrating radiations (13). In contrast to DSA, DVA does not use a mask but calculates the standard deviation, variance, and other time-derived parameters of X-ray attenuation for each pixel in an unsubtracted image series. Thereby, DVA enhances the moving contrast media–related signal (ie, the flow of contrast agents) and suppresses the noise, resulting in improved image quality. DVA has previously been shown to be superior to DSA in nonselective ICM lower limb interventions, in ICM studies with metal objects, and in nonselective lower limb interventions using CO_2_ as the contrast agent (14, 15, 16, 17). Apart from the standard DVA algorithm (DVA1), another DVA algorithm (DVA2) has recently been developed, allowing additional noise reduction. DVA2 was developed with the purpose of achieving high-quality diagnostic imaging at a lower radiation and/or ICM dose setting than what is currently achievable with DVA1.

Based on the reported noise reduction and improved image quality of DVA in nonselective lower limb angiography, the aim of the current study was to investigate whether the quality advantages of this new image processing technology could also be observed in selective lower limb interventions, where the image quality of DSA is usually higher due to the administration of contrast agents closer to the location of lesions. The secondary aim of the study was to compare the performance of 2 different DVA algorithms in such a clinical setting.

## Materials and Methods

### Patient Selection

Of a total of 28 patients with peripheral artery disease (Fontaine Stages II to IV), 15 patients (mean age, 72.6 years; range, 54–88 years) underwent interventions using ICM and 13 patients (mean age, 77.25 years; range, 52–89 years) underwent interventions using CO_2_ from November 2019 to May 2020, and were included in this retrospective observational study. Four patients underwent angiography using both CO_2_ and ICM during different interventions. One patient who received ICM underwent successive interventions at 3 different episodes. The ICM subgroup consisted of 6 men (mean age, 69 years; range, 54–81 years) and 9 women (mean age, 75 years; range, 63–88 years). The CO_2_ group consisted of 6 men (mean age, 78.8 years; range, 64–85 years) and 7 women (mean age, 77.4 years; range, 52–89 years).

### Study Design

For the ICM patient group, images were acquired before and after the intervention in 3 anatomical regions (femoral, popliteal, and talocrural), and at least 2 image series acquired before and after the intervention were included in the analysis. With CO_2_ angiography, problems were often encountered during the acquisition of images; many patients did not tolerate the injection of CO_2_ because of unbearable pain despite analgesics, and the angiography was continued with conventional ICM. Therefore, in the CO_2_ group, 1 image series acquired in any anatomical region before and after the intervention was set as the minimum. Only those acquisitions were omitted where the DSA image was not appropriate for diagnostic evaluation. DSA, DVA1, and DVA2 images were compared in terms of the contrast-to-noise ratio (CNR) values and visual image quality. All the evaluations and calculations were done retrospectively. An approval from the institutional review board was not required for this type of retrospective study.

### Image Acquisition

The femoral artery was chosen as the standard vascular access for all angiographic procedures. An interventional radiologist (R.P.T.) with more than 14 years of experience in interventional procedures performed the angiography in all cases. For angiography using ICM, the ICM was injected manually intra-arterially. Angiography using CO_2_ angiography was performed using an automated CO_2_ delivery system with controlled pressures (Angiodroid; Angiodroid, San Lazzaro, Italy). The indication for CO_2_ angiography included renal impairment (reflected by serum creatinine values of >1.3 mg/dL and glomerular filtration rate of <55 mL/min), previous history of ICM allergy, or renal transplantation. Antegrade or retrograde crossover approaches were decided by the operator, depending on the location of the pathology. A 5-F catheter sheath was used for the diagnostic angiograms, whereas a 6-F access was used for interventions such as balloon angioplasties and stent implantations. ICM (Ultravist 300; Bayer Vital GmbH, Leverkusen, Germany) volume, with a common dilution of 3:2 with physiological saline, was determined for each patient. All procedures were performed in the angiography suite (30 × 40-cm detector, Siemens Artis zee; Siemens Healthineers, Erlangen, Germany) with standard image acquisition protocols for lower limb angiography (2 frames/sec). DSA images during CO_2_ angiography were acquired using predefined protocols specifically developed for CO_2_ imaging (Evenflow [frame rate of 7.5/sec]; Siemens Healthineers). The acquired unsubtracted series was used for the generation of both DSA and DVA images.

### Image Processing

Postprocessed DSA images were generated during or immediately following the acquisition using Syngo software (Siemens Healthineers). The DVA images were generated retrospectively from the same raw (unsubtracted) angiographic image series using the Kinepict Medical Imaging Tool (Kinepict Health, Budapest, Hungary). Both the processing methods included respective motion correction (pixel shift) and brightness/contrast adjustments. All images were stored as digital imaging and communications in medicine (DICOM) files for CNR analysis and converted to lossless tagged image format files (TIFF) for web-based visual evaluation.

### CNR Analysis

For CNR calculations, regions of interest (ROI) were defined on the vessels and the background regions using ImageJ (18). The ROIs were placed in pairs: 1 vascular ROI and 1 adjacent background ROI. The angiographic images were categorized into 3 regions: femoral, including the hip joint; popliteal, including the knee joint; and talocrural, below the knee. Three vascular and extravascular pairs of ROIs were placed on every large vascular section. The ROIs placed on the DVA images were readjusted to the corresponding DSA image when there was any geometric difference between the images due to pixel shifting. CNR was calculated for all the ROI pairs individually according to the following formula:

$$CNR=\;\frac{\left|Mean_{v}-Mean_{b}\right|}{Std_{b}},$$

where $\;Mean_{v}$ and $Mean_{b}$ refer to the mean pixel intensity values of the vascular and background ROI, respectively, and $Std_{b}$ is the background standard deviation.

CNR of the corresponding DVA and DSA ROIs were calculated. Using the CNR data, the ratio of CNR values in different groups (R), that is, CNR_DVA(1/2)_/CNR_DSA_ and CNR_DVA2_/CNR_DVA1_, were also calculated.

### Qualitative Comparison

The visual evaluation was performed by 6 radiologists (R.P.T., S.V., A.M.K., S.S.A., O.E., and J.S.) with 7–14 years of interventional experience from 2 different institutions (3 from each). A randomized blinded web-based survey was created, and the images were evaluated using a 5-grade Likert scale based on the visibility and diagnostic value of large and small arteries. The images were graded according to the following scores: 1 = poor image quality (unsuitable for diagnosis); 2 = low image quality (main vessels are distinguishable but not examinable); 3 = medium image quality (the main vessels are examinable, but the diagnosis of vessels with a diameter of <2.5 mm is questionable); 4 = good image quality (even the smaller vessels are examinable; suitable for everyday use); and 5 = outstanding image quality (much richer in details than the everyday routine; facilitating optimal clinical decision-making).

The fully anonymized images appeared in random order without disclosure of the image type. The scores were collected in a database for later processing. The ICM and CO_2_ images were grouped into 2 separate surveys because of the obvious differences in appearance and image quality.

### Statistical Analysis

Microsoft Excel (Microsoft, Redmond, Washington) was used for the analysis of CNR and for the calculation of medians and confidence intervals. Statistical analyses were performed using SPSS version 25 (IBM, Armonk, New York). A *P* value of <.05 was considered significant. The evaluators scored the individual image sets from 1 to 5 as described above. The mean and standard error of the mean were calculated. Interquartile ranges and medians were additionally determined because of the asymmetric dispersions of data. Paired groups were analyzed using the Wilcoxon signed-rank test.

## Results

### CNR Measurements

From the 112 ICM and 40 CO_2_ images (DSA, DVA1, and DVA2), 4,742 and 852 ROIs were manually selected and used for CNR calculations.

#### DVA versus DSA

**Figure 1:**
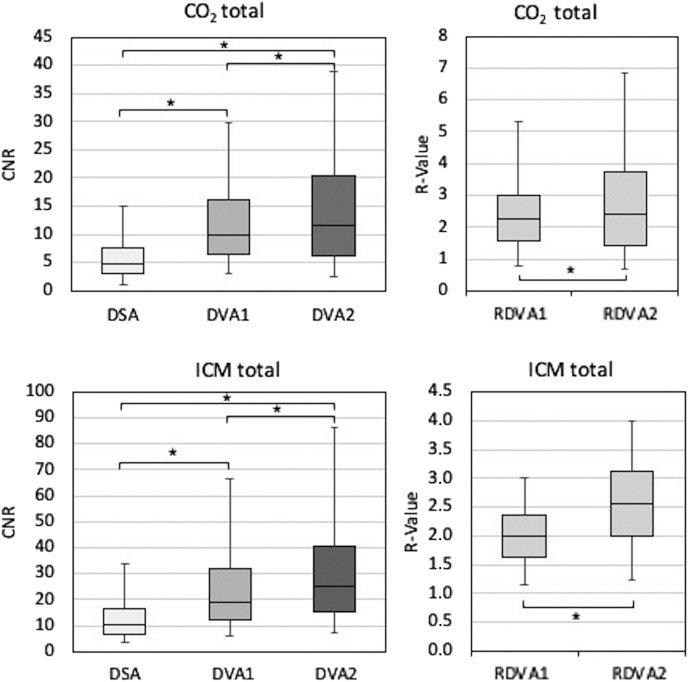
Contrast-to-noise ratio (CNR) comparison in 40 carbon dioxide (CO_2_) and 112 iodinated contrast media (ICM) digital subtraction angiography (DSA), digital variance angiography algorithm 1 (DVA1), and digital variance angiography algorithm 2 (DVA2) image pairs. R value of DVA1 and DVA2 compared with DSA. The box and whisker plots show the median (line), interquartile range (box), and internal fences (whiskers) of CNR values in each group and R values, respectively. RDVA1 = R value of DVA1/DSA; RDVA2 = R value of DVA2/DSA. ∗*P* < .05 (Wilcoxon signed-rank test).

The DVA images, independent of the algorithm and contrast agent used, provided significantly higher CNR values than the DSA images (*P* < .001) (Table 1). Depending on the anatomical region, the median R value for the ICM images ranged from 1.90 to 2.08 (overall median, 1.98) for DVA1 and from 2.44 to 2.63 (overall median, 2.55) for DVA2 (Fig 1). For CO_2_ images, the median R value ranged from 1.84 to 2.71 (overall median, 2.24) for DVA1 and from 1.79 to 2.76 (overall median, 2.42) for DVA2 (Fig 1).

**Table 1:** Contrast-to-Noise Ratio Measurements

Contrast agent	Region (No. of ROI)	CNR values			R values		
		DSA	DVA1	DVA2	DVA1/DSA	DVA2/DSA	DVA2/DVA1
ICM	Femoral (n = 1792)	14.4 (8.7–23.3)	25.1 (15.3–44.4)	31.4 (18.9–55.5)	1.90 (1.57–2.25)	2.44 (1.92–2.90)	1.26 (1.05–1.51)
	Popliteal (n = 730)	11.3 (7.4–16.6)	22.2 (13.9–35.2)	28.0 (17.4–42.6)	2.08 (1.76–2.43)	2.60 (1.94–3.25)	1.26 (1.05–1.51)
	Talocrural (n = 2220)	8.0 (5.2–12.1)	15.9 (10.1–24.7)	20.1 (12.9–31.3)	2.01 (1.70–2.39)	2.63 (2.06–3.22)	1.32 (1.03–1.61)
	All (N = 4742)	10.2 (6.3–16.4)	19.2 (12.0–32.1)	25.2 (15.0–40.7)	1.98 (1.64–2.36)	2.55 (1.99–3.1)	1.28 (1.05–1.55)
CO_2_	Femoral (n = 476)	5.4 (3.1–8.0)	11.1 (7.3–17.8)	14.3 (8.4–24.5)	2.35 (1.15–3.03)	2.76 (1.65–4.20)	1.12 (0.89–1.72)
	Popliteal (n = 154)	3.6 (1.7–9.5)	8.3 (4.9–16.5)	7.7 (3.2–17.4)	2.71 (1.87–3.44)	2.17 (1.41–3.07)	0.86 (0.59–1.13)
	Talocrural (n = 222)	4.6 (3.7–5.9)	8.5 (6.4–11.1)	9.0 (5.3–13.5)	1.84 (1.52–2.36)	1.79 (1.07–3.31)	1.02 (0.68–1.53)
	All (N = 852)	4.87 (3.01–7.65)	9.8 (6.5–16.1)	11.58 (6.26–20.5)	2.24 (1.57–3.0)	2.42 (1.43–3.76)	1.11 (0.76–1.57)

Note–Median CNR values (with interquartile Q1–Q3 ranges) for DSA, DVA1, and DVA2, and the respective R values in the femoral, popliteal, and talocrural region. Wilcoxon signed-rank test showed significant differences (*P* < .05) in all paired comparisons of the CNR values, except in the CO_2_ talocrural DVA2 versus DVA1 comparison, where the *P* value was .088.

ICM= iodinated contrast media; CNR = contrast-to-noise ratio; CO_2_ = carbon dioxide; DSA = digital subtraction angiography; DVA1 = digital variance angiography algorithm 1; DVA2 = digital variance angiography algorithm 2; ICM = iodinated contrast media; ROI = region of interest.

#### DVA1 versus DVA2

For ICM images, DVA2 provided a slightly higher CNR (median DVA2/DVA1 range, 1.26–1.32; overall median, 1.28), but the difference was negligible for CO_2_ images (median range, 0.86–1.12; overall median, 1.11) (Table 1).

### Visual Evaluation

**Figure 2:**
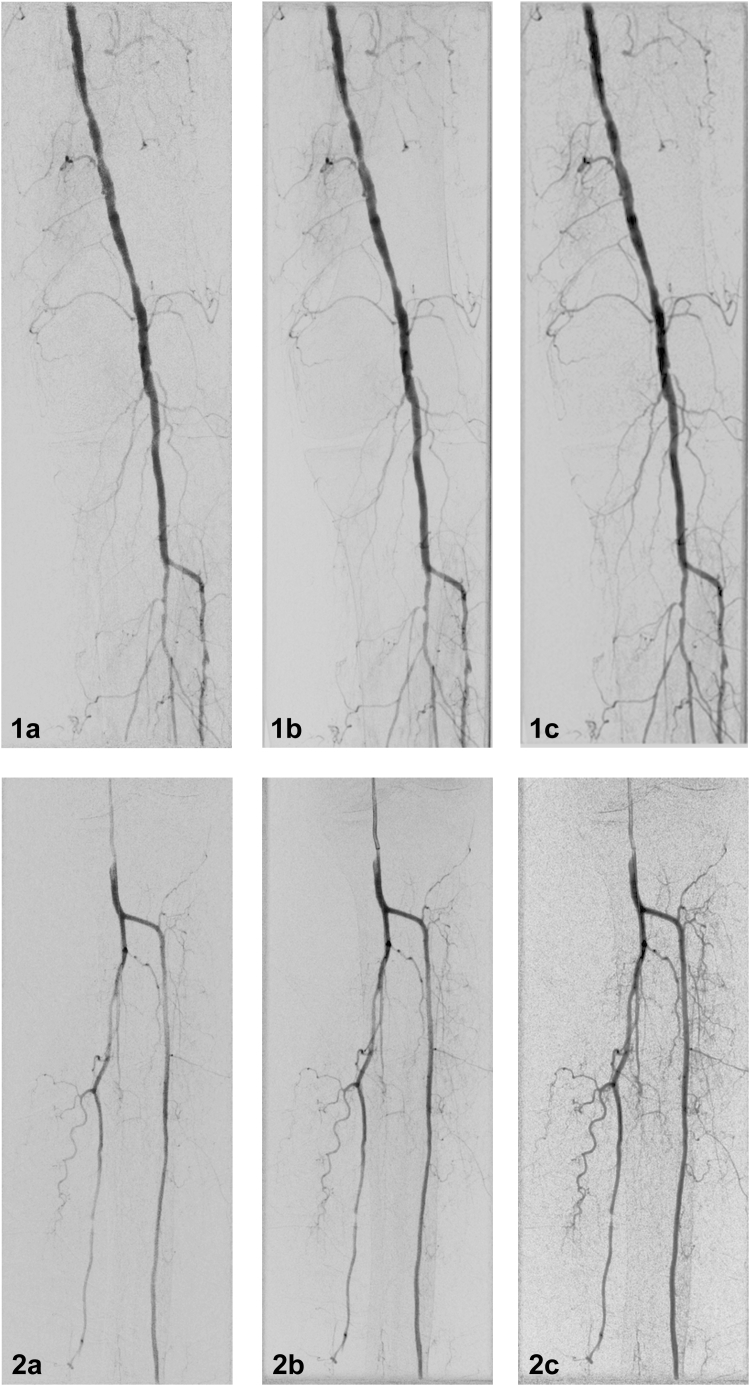
Iodinated contrast media representative image pairs in popliteal (1) and talocrural (2) regions. **(a)** Digital subtraction angiography. **(b)** Digital variance angiography algorithm 1. **(c)** Digital variance angiography algorithm 2.

Altogether, 112 ICM and 40 CO_2_ acquisitions were included in the visual evaluation. DSA, DVA1, and DVA2 images were generated from each unsubtracted series. In total, the evaluators rated 336 ICM and 120 CO_2_ images of different types in a blinded and randomized manner. Representative image triplets generated from the same raw acquisition are shown in Figures 2a–c (ICM) and 3a–c (CO_2_).

**Figure 3:**
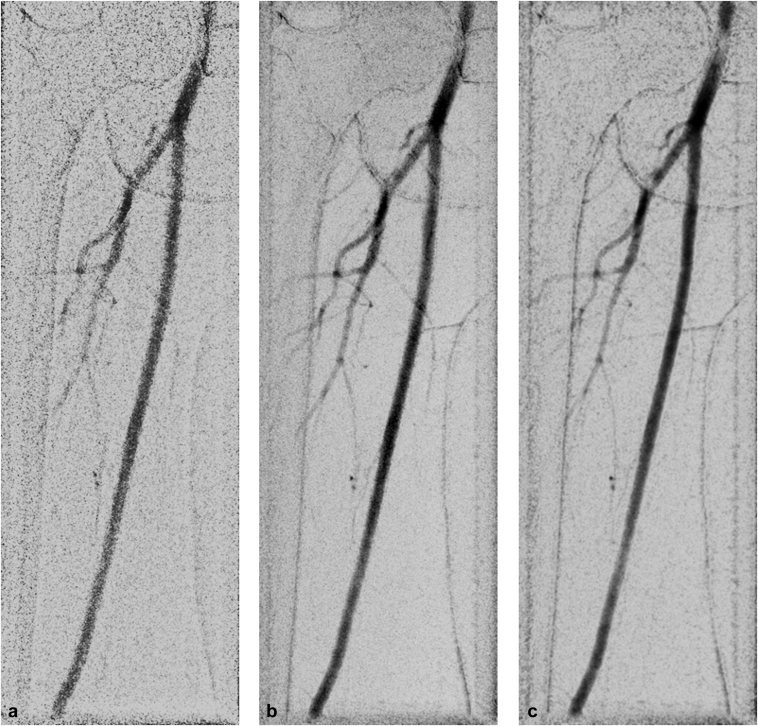
Carbon dioxide representative image pairs in the femoral region. **(a)** Digital subtraction angiography. **(b)** Digital variance angiography algorithm 1. **(c)** Digital variance angiography algorithm 2.

#### DVA versus DSA

**Figure 4:**
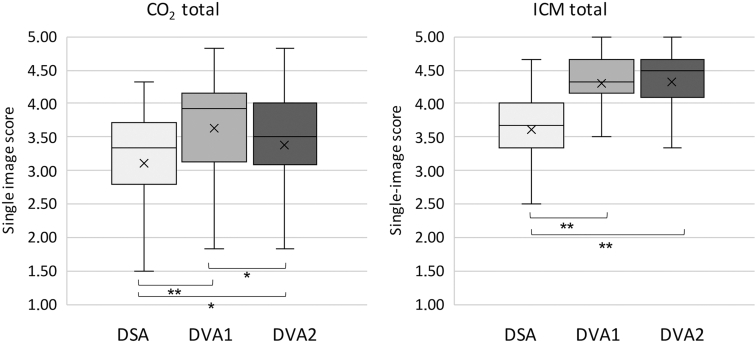
Single image evaluation score comparison of 40 carbon dioxide (CO_2_) and 112 iodinated contrast media (ICM) digital subtraction angiography (DSA), digital variance angiography algorithm 1 (DVA1), and digital variance angiography algorithm 2 (DVA2) image pairs rated by 6 experts. The box and whisker plots show the median (line), mean (x), interquartile range (box), and internal fences (whiskers). ∗*P***<** .05, ∗∗*P* < .001 (Wilcoxon signed-rank test).

In the ICM group, both DVA1 and DVA2 provided significantly higher image scores than DSA in all subregions and in overall comparison (Table 2). The overall scores (expressed as mean ± standard error of the mean) were 3.61 ± 0.05 for DSA, 4.30 ± 0.04 for DVA1 (*P* < .001 vs DSA), and 4.33 ± 0.04 for DVA2 (*P* < .001 vs DSA) (Fig 4). The lowest difference in DVA and DSA means was found in the femoral region (+0.56 and +0.59 for DVA1 and DVA2, respectively), whereas the highest difference was observed in the crural region (+0.73 and +0.80 for DVA1 and DVA2, respectively).

**Table 2:** Visual Evaluation Data for Iodinated Contrast Media

Processing method	Mean	SEM	Median	Q1–Q3	DSA vs DVA	DVA1 vs DVA2
Femoral (n = 37)						
DSA	3.93	0.04	4.00	3.83–4.00		
DVA1	4.49	0.05	4.50	4.33–4.67	*P* < .001	*P* = .757
DVA2	4.52	0.04	4.50	4.33–4.67	*P* < .001	
Popliteal (n = 23)						
DSA	3.64	0.11	3.67	3.67–3.83		
DVA1	4.31	0.08	4.33	4.25–4.58	*P* < .001	*P* = .834
DVA2	4.31	0.07	4.33	4.08–4.50	*P* < .001	
Crural (n = 52)						
DSA	3.40	0.08	3.50	3.00–3.83		
DVA1	4.13	0.08	4.25	3.83–4.50	*P* < .001	*P* = .06
DVA2	4.20	0.07	4.33	3.83–4.54	*P* < .001	
Overall (N = 112)						
DSA	3.61	0.05	3.67	3.33–4.00		
DVA1	4.30	0.04	4.33	4.16–4.66	*P* < .001	*P* = .169
DVA2	4.33	0.04	4.50	4.08–4.66	*P* < .001	

Note–Individual ICM image evaluation was conducted using a 5-grade Likert scale by 6 experienced radiologists. Wilcoxon signed-rank test (*P* < .05) was performed comparing DSA, DVA1, and DVA2. Q1–Q3 refers to the interquartile range.

DSA = digital subtraction angiography; DVA = digital variance angiography; DVA1 = digital variance angiography algorithm 1; DVA2 = digital variance angiography algorithm 2; ICM = iodinated contrast media; SEM = standard error of mean.

In the CO_2_ group, the overall DVA values were also significantly higher than the DSA scores (3.10 ± 0.14 for DSA; 3.63 ± 0.13 for DVA1, *P* < .001; 3.38 ± 0.13 for DVA2, *P* < .05) (Fig 4). However, in the femoral, popliteal, and crural regions, only the DVA1 scores proved to be significantly higher than the DSA values. There was no significant difference between the DSA and DVA2 scores (Table 3), although the mean DVA2 scores were consistently higher. In the subregions, DVA1 followed the same pattern as in the ICM group. The lowest difference was observed in the femoral (+0.47) region, and the highest difference was observed in the crural (+0.64) region. On the other hand, DVA2 performed better in the femoral (+0.35) than in the crural (+0.15) region.

**Table 3:** Visual Evaluation Data for Carbon Dioxide Contrast

Processing method	Mean	SEM	Median	Q1–Q3	DSA vs DVA	DVA1 vs DVA2
Femoral (n = 22)						
DSA	3.07	0.18	3.25	2.88–3.67		
DVA1	3.54	0.19	3.75	3.21–4.17	*P* = .033	*P* = .147
DVA2	3.42	0.18	3.58	3.21–3.21	*P* = .056	
Popliteal (n = 7)						
DSA	3.19	0.38	3.67	2.58–3.92		
DVA1	3.74	0.34	4.00	3.25–4.25	*P* = .026	*P* = .115
DVA2	3.40	0.20	3.50	3.17–3.83	*P* = .345	
Crural (n = 11)						
DSA	3.12	0.33	3.33	2.50–3.75		
DVA1	3.76	0.28	4.00	3.25–4.33	*P* = .003	*P* = .021
DVA2	3.27	0.35	3.33	2.92–3.83	*P* = .305	
Overall (N = 40)						
DSA	3.10	0.14	3.33	2.79–3.71		
DVA1	3.63	0.13	3.92	3.13–4.17	*P* < .001	*P* = .001
DVA2	3.38	0.13	3.50	3.01–4.00	*P* = .022	

Note–Individual CO_2_ image evaluation was conducted using a 5-grade Likert scale by 6 experienced radiologists. Wilcoxon signed-rank test (*P* < .05) was performed comparing DSA, DVA1, and DVA2. Q1–Q3 refers to the interquartile range.

CO_2_ = carbon dioxide; DSA = digital subtraction angiography; DVA = digital variance angiography; DVA1 = digital variance angiography algorithm 1; DVA2 = digital variance angiography algorithm 2; SEM = standard error of mean.

#### DVA1 versus DVA2

In the ICM group, there was no significant difference between the DVA1 and DVA2 scores, and the performance was indistinguishable; the difference in the means of DVA2 and DVA1 ranged between 0.00 and 0.07. Yet for CO_2_, DVA1 proved to be significantly better in the crural region and in the overall evaluation; the difference in the means of DVA2 and DVA1 ranged between −0.12 and −0.49.

## Discussion

Previous studies (14,16) have reported that DVA provides higher quality images in nonselective lower limb angiography, where the contrast agent is injected infrarenally above the aortic bifurcation. The main aim of this study was to investigate whether this quality advantage can also be observed with different DVA algorithms (DVA1 and DVA2) in selective lower limb interventions. In this study, the contrast agent (ICM or CO_2_) was injected into the femoral or popliteal arteries closer to the site of the lesion, and thereby the obtained DSA image had higher quality than in nonselective settings. The CNR values, the objective predictors of image quality, clearly verified the advantage of DVA technology over DSA. Regardless of the anatomical region, contrast agent, and DVA algorithm, the DVA images delivered almost 2-fold or higher CNR values than the DSA images. Between the 2 different DVA algorithms (DVA1 and DVA2), the latter provided approximately 30% higher CNR for the ICM acquisitions; however, there was no major difference in the CNR values for the CO_2_ images, where DVA2 provided only 11% higher CNR than DVA1.

The visual evaluation data in general corresponded to the CNR measurements, although the correlation was not perfect - that is, a higher CNR did not always result in better subjective image quality. In the ICM group, both DVA algorithms provided significantly higher visual scores than DSA, with the most visible difference in the talocrural region (0.73 and 0.80 for DVA1 and DVA2, respectively). This is an important clinical difference considering the grading scale: Grade 3 represents a limited diagnostic value suitable only for discrimination of the main arteries, whereas Grade 4 represents the full diagnostic capacity covering the small arteries as well. In the CO_2_ group, however, only DVA1 provided consistently and significantly higher scores, whereas DVA2 was significantly higher than DSA only in the overall comparison. Although DVA2 had higher CNRs than DVA1, there was no difference in the visual scores of the DVA algorithms in most comparisons. Moreover, the overall DVA1 score in the CO_2_ group was significantly higher than the DVA2 score.

The objective CNR and subjective visual evaluation data clearly show that DVA, independent of the contrast agent used, has a quality advantage not only in nonselective but also in selective lower limb angiography settings, which corresponds to the daily routine setting in interventional procedures (14,16). The improved image quality may expedite or increase confidence in the decision making process during interventional procedures. At the same time, the number of repeated contrast medium injections in the procedural decision-making could be reduced, especially in CO_2_ angiography, where the image quality is lower than with ICM angiography. Another possible advantage of the observed quality advantage is the potential for radiation dose and contrast medium dose management. A recent paper (19) reported that DVA allows 50% ICM reduction in carotid angiography, without compromising the image quality. Another preliminary report (20) indicates that the quality advantage can also be used for a substantial reduction (approximately 70%) of DSA-related radiation exposure in lower limb angiography. This dose management capability of DVA could therefore contribute enormously to the safety of interventions for both the patients and the medical personnel, without compromising the image quality. With all these advantages, DVA has substantial potential to be developed as an effective tool for all minimally invasive endovascular interventions in the future.

With regard to the comparison of the different DVA algorithms, the results of this study showed that although DVA2 provided moderately higher CNR than DVA1, its subjective image quality was not better. Moreover, DVA1 received significantly higher scores in the CO_2_ group. DVA2 was developed for improving the performance of DVA technology in dose management settings, that is, when either the radiation dose or the volume of contrast agent is reduced. Under these conditions, the image quality is weaker and usually the noise is higher than under the normal conditions. DVA2 has an improved noise filter, which indeed can increase the CNR; however, its processing time is longer (2–8 sec, depending on the file size), whereas DVA1 is generated typically within 1 second. Since DVA2 could not provide better image quality in this study, the standard DVA1 algorithm is recommended for selective lower limb angiographies. Nevertheless, it should be noted that all the interventions were performed with normal settings (in terms of radiation dose and contrast media volume); therefore, the evaluation of the potential use of DVA2 in real dose management settings needs to be studied in the future.

Although CNR is considered an objective measure of image quality, the data indicate that CNR cannot be directly translated to subjective image quality, since a higher CNR (30% difference) did not result in better perceived image quality (see overall DVA1 vs DVA2 in the ICM group), or a similar CNR did not result in similar perceived image quality (see overall DVA1 vs DVA2 in the CO_2_ group). This indicates that the use of CNR data alone are not likely to be sufficient to characterize the image quality.

The study has some limitations. The number of patients is relatively low, but a small cohort proof-of-concept study is the first step. Despite the low number of patients, especially in the CO_2_ group, where some anatomical regions were underrepresented, significant differences were observed. Because of strong motion artifacts, several images were excluded, mainly in the CO_2_ group; however, only those acquisitions were excluded where the DSA image was not suitable for diagnosis even after the best available postprocessing steps (eg, remasking, pixel shifting). Thus, the prepared DVA images were compared with all clinically evaluable DSA images. The study performed retrospective image analyses, and based on the positive results, larger scale multicentric prospective studies could be warranted. Real-time prospective analysis should be used to validate the diagnostic and interventional advantages of DVA. Installing DVA technology in the procedure room enables real-time data processing, which was recently reported to be helpful in CO_2_-assisted lower limb interventions (17).

In conclusion, the current study provides evidence that DVA offers significantly better image quality than DSA in selective lower limb angiography using either ICM or CO_2_ as a contrast agent. Furthermore, the originally developed DVA1 algorithm is the preferred choice in this clinical setting. The observed quality advantage might provide an opportunity for the reduction of radiation and/or contrast agent dose, and thereby, the safety of interventions could be enhanced for both the patients and the medical staff. Nevertheless, these potential benefits of DVA need to be validated in larger scale prospective multicentric studies.
